# Mst1 Directs Myosin IIa Partitioning of Low and Higher Affinity Integrins during T Cell Migration

**DOI:** 10.1371/journal.pone.0105561

**Published:** 2014-08-18

**Authors:** Xiaolu Xu, Emily R. Jaeger, Xinxin Wang, Erica Lagler-Ferrez, Serge Batalov, Nancy L. Mathis, Tim Wiltshire, John R. Walker, Michael P. Cooke, Karsten Sauer, Yina H. Huang

**Affiliations:** 1 Department of Pathology and Immunology, Washington University, St. Louis, Missouri, United States of America; 2 Genomics Institute of the Novartis Research Foundation, San Diego, California, United States of America; 3 Division of Pharmacotherapy and Experimental Therapeutics, Eshelman School of Pharmacy, University of North Carolina, Chapel Hill, North Carolina, United States of America; 4 Department of Immunology and Microbial Science, The Scripps Research Institute, La Jolla, California, United States of America; 5 Departments of Pathology and Microbiology & Immunology, The Geisel School of Medicine at Dartmouth, Lebanon, New Hampshire, United States of America; Leiden University, Netherlands

## Abstract

Chemokines promote T cell migration by transmitting signals that induce T cell polarization and integrin activation and adhesion. Mst1 kinase is a key signal mediator required for both of these processes; however, its molecular mechanism remains unclear. Here, we present a mouse model in which Mst1 function is disrupted by a hypomorphic mutation. Microscopic analysis of *Mst1*-deficient CD4 T cells revealed a necessary role for Mst1 in controlling the localization and activity of Myosin IIa, a molecular motor that moves along actin filaments. Using affinity specific LFA-1 antibodies, we identified a requirement for Myosin IIa-dependent contraction in the precise spatial distribution of low and higher affinity LFA-1 on the membrane of migrating T cells. *Mst1* deficiency or Myosin inhibition resulted in multipolar cells, difficulties in uropod detachment and mis-localization of low affinity LFA-1. Thus, Mst1 regulates Myosin IIa dynamics to organize high and low affinity LFA-1 to the anterior and posterior membrane during T cell migration.

## Introduction

Human mutations in the *Mst1* gene result in a primary immunodeficiency disease [1]–[3]. Affected patients experience recurrent viral and bacterial respiratory infections as well as cutaneous lesions resulting from Human Papillomavirus infections. Defective immune protection against these infections is due to T cell deficiency [1]–[3]. *In vivo* and *in vitro* analyses of *Mst1* deficient mice have been instrumental in identifying Mst1 as a key regulator of T cell trafficking [4]–[7]. The ability of T cells to continually circulate through the body is critical for immune protection (reviewed in [8]).

Different T cell subsets have distinct trafficking patterns. Naïve and central memory T cells traffic between the blood and lymphatics. They patrol secondary lymphoid organs such as the spleen and lymph nodes for cognate antigen brought there by tissue-derived antigen presenting cells. In contrast, effector T cells traffic to and within inflamed tissue to promote inflammation and mediate direct target cell killing. T cell trafficking patterns are programmed by the expression of membrane chemokine receptors and adhesion molecules, including selectins and integrins [9]. T cells enter secondary lymphoid organs and peripheral tissue from the vasculature by extravasation. Selectins mediate T cell rolling along the endothelium while integrins provide the strong adhesion required for stopping and squeezing through the endothelium. Within the lymph node, naïve and central memory T cells are guided by the chemokines CCL19 and CCL21 to migrate along fibroblastic reticular cells in an integrin-independent manner. In the absence of antigen, T cells leave the lymph nodes via the lymphatics to downstream lymph nodes and eventually return to the blood. Similarly, effector T cells are recruited to sites of infection by chemotactic cues and extravasate in an integrin-dependent manner. However, unlike within lymphoid organs, inflammation restructures the peripheral tissue environment and upregulate integrin ligands [10]. Migration of effector T cells within the inflamed tissue is highly dependent on integrins and is completely disrupted by integrin blocking antibodies [10].

T cell responses to chemokines and integrin activation are critical for migration. Chemokines induce T cell polarization and impart migratory directionality. Integrins mediate adhesion and extravasation through endothelia. Mst1 differentially regulates these processes. *Mst1* deficient T cells show defects in CCL19-induced polarization *in vitro* and decreased migratory velocity within lymph nodes and thymus [6], [11]. *Mst1* deficiency also leads to significant defects in T cell egress from the thymus and in lymph node entry, demonstrating that Mst1 function is required for extravasation [4]–[7], [11]. *In vitro* analysis of adhesion show that while selectin-dependent rolling is unaffected, integrin-dependent firm adhesion is Mst1-dependent [6].

Integrin-mediated adhesion is a highly regulated process. Integrin affinity and avidity are increased by inside-out signaling downstream of the T cell receptor (TCR) or chemokine receptor (CCR) [12]. Inside-out signaling changes the orientations of the cytoplasmic tails of integrin alpha and beta chains to allow the extracellular domains to adopt higher affinity conformations [13]. In addition, binding avidity increases through clustering of multiple LFA-1 receptors. Activation of the small GTPase Rap1 mediates both increased integrin affinity and avidity [14]. Recently, separate Rap1 effector complexes were identified to associate with the cytoplasmic domains of LFA-1 subunits. RapL binds directly to the αL subunit (CD11a) while RIAM in association with Kindlin-3 binds to the β2 subunit (CD18) [15]. Both RapL and RIAM complexes contain Mst1 and are dependent on ADAP/SKAP55 adapter proteins [15], suggesting that Mst1 may contribute to affinity and avidity maturation. However, ICAM-1-Fc fusion proteins equally stain wt and *Mst1* deficient T cells, indicating that LFA-1 affinity activation is Mst1-independent [5]. In contrast, *Mst1*-deficient T cells show defects in global LFA-1 clustering [5]. This indicates that Mst1 participates in inside-out signaling to regulate integrin clustering, although the underlying molecular mechanism remains elusive.

Integrin affinity differs among topological locations on the membrane of migrating T cells. LFA-1 molecules at the leading edge and midbody are in the intermediate and high affinity conformations, respectively, while uropodal and trailing edge LFA-1 molecules have low ligand affinity [16]–[18]. This allows the leading edge to form nascent adhesive contacts [18], the midbody to firmly adhere to establish traction and the trailing edge to detach from the substratum [17]. Although Mst1 does not regulate LFA-1 affinity maturation [5], it remains to be determined whether Mst1 controls the distribution of different affinity LFA-1 molecules.

The actinomyosin contractile module is a well-studied mechanotransduction machine that regulates integrin-dependent and independent migration [19]. The ATP-dependent motor protein, Myosin IIA generates force on filamentous (F-) actin to induce T cell contraction. Myosin-mediated contraction is necessary for the establishment of new adhesion at the lamellipodium [20], [21] and the detachment of low affinity integrins at the uropod [21], [22]. Myosin is also important for integrin-independent migration in interstitial tissue via a cyclical squeezing and pushing mode of movement [23], [24]. And, more broadly Myosin is required for the maintenance of cell polarity and morphology. Myosin-IIa deficient or inhibited cells are either unable to polarize or become multipolar [25], [26] and are severely defective in migration through intact endothelium and small pores requiring cellular contractility [27], [28]. Here, we demonstrate that Myosin IIa localization is disrupted in *Mst1*-deficient T cells, suggesting that Mst1 acts upstream of Myosin. Interestingly, in epithelial cells, the actinomyosin module can activate transcription via YAP/TAZ, a downstream inhibitory target of the Mst1/Hippo pathway [29]. However, YAP/TAZ activation in this model appears to be Mst1-independent [29]. We report a new role for Myosin IIa in controlling adhesion through the proper spatial distribution of low and high affinity LFA-1 during T cell migration. Additionally, we show that Mst1 acts through Myosin IIa to regulate polarization and adhesion during migration.

## Materials and Methods

### Mice

*Mst1^h/h^* (*WeeT*) mice were identified by flow cytometric screening of peripheral blood of G3 progeny from C57BL/6 male mice treated with *N*-ethyl-*N*-nitrosourea (ENU) as previously described [30], [31]. For phenotypic analysis and mechanistic studies, *Mst1^h/h^* mice were backcrossed to wt C57BL/6 mice for 10 generations to remove other ENU-induced mutations. To identify the causative mutation in *WeeT* mice, affected C57BL/6 mice were bred to 129Sv/ImJ mice to generate hybrid F2 mice for mapping. Single nucleotide polymorphism (SNP) assays across the entire genome (n = 356) were performed using the Sequenom MassARRAY system [32]. Map Manager QTX was used to calculate logarithm of the odds (LOD) scores and perform interval mapping [33]. Sequencing was performed on an Illumina genome analyzer after enriching genomic DNA for the mapped region using a custom Nimblegen array (Short Read Archive # SRA059354). Mice were housed in a specific pathogen-free facility. Experimental protocols were approved by the GNF Animal Study Committee, the Washington University Animal Study Committee (protocol #20110133) and the Dartmouth College Institutional Animal Care and Use Committee (protocol # huan.yh.1).

### Detection of Mst1 transcripts and protein

For *Mst1* mRNA detection, cDNA was synthesized from T cells with SuperScript II Reverse Transcription kit (Invitrogen). Quantitative PCR was carried out using SYBR Green Master Mix (Agilent) on a PRISM 7000 Sequence Detection System (Applied Biosystems) using GAPDH and *Mst1* primers: 5′-GCAGGCAGCTGAAAAAGTT-3′ and 5′-CCATAAGACCCCTCTCCAAG-3′. For Mst1 protein detection, purified CD4^+^ T cells (Invitrogen) treated with vehicle, MG132 or Z-DEVD were lysed with Triton X-100 lysis buffer ((1% Triton X-100, 50 mM Tris pH 8.0, 100 mM NaCl, proteases inhibitors (Roche). Pre-cleared cell lysates were analyzed by western blot analysis with Mst1 (Cell Signaling) and beta-actin (Sigma) antibodies.

### Cell staining

For flow cytometric analysis, cells were stained with antibodies against CD8-PE/Cy7, CD45.2-APC750, CD45.1-PerCP/Cy5.5 (eBioscience), Vα11-FITC, CD24-PE, CD62L-Pacific Blue, CD69-PE, CCR7-APC, CD4-APC, (Biolegend). For LFA-1 localization, purified CD4^+^ T cells were stained with anti-CD44-Alexa488 and anti-CD11a-Alexa647 (M17/4)) and fixed with 4% paraformaldehyde. For live imaging, staining with anti-CD11a-Alexa647 (M17/4)), and anti-CD11a-Alexa546 (2D7) was performed during imaging, at a concentration of 0.08 ng/mL to prevent integrin blockade.

### Confocal and TIRF Microscopy and Image Analysis

Images were captured under a FluoView-1000 laser-scanning confocal microscope (Olympus). For live imaging, cells were kept in Leibovitz's L-15 buffer (Gibco) supplemented with 2% FCS. Captured images and videos were preprocessed in ImageJ (NIH) and analyzed using MATLAB (MathWorks) to detect individual cells and quantify clustering of fluorescently tagged proteins. Single cell detection was performed with custom-built software written in MATLAB. Clustering of protein was quantified on singularly detected cells as described previously [34]. Briefly, mean pairwise distance of the pixels of the top 10% intensity was calculated as *Dα*. *S_l_* was the mean pairwise distance of the same number of pixels packed together as a 10 by 10 pixel square, as the upper limit of the clustering. *S_u_* was the mean pairwise distance of the same number of pixels uniformly scattered on the cell perimeter, as the lower limit of the clustering. A clustering index was calculated using the following equation:
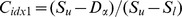



### Transwell Assay

Purified CD4 T cells were seeded into top chambers over 3 μm or 5 μm transwell filters with 100 ng/mL CCL19 (PeproTech) in the bottom chamber. After 1.5 hrs at 37°C, cells were recovered from the lower chamber and counted by high throughput enabled flow cytometer LSR II (BD). Percentage of migrated cells was determined as a percentage of total input. In some cases, the transwell filters were pre-coated with BSA or 2 μg/mL ICAM-1-Fc (R&D Systems).

### Statistics

GraphPad Prism was used to perform Student's t-test on normally distributed data and Mann-Whitney or Wilcoxon ranked sum test for non-normally distributed data.

## Results

### *WeeT* mice are T cell deficient due to a mutation in *Mst1*

**Figure 1:**
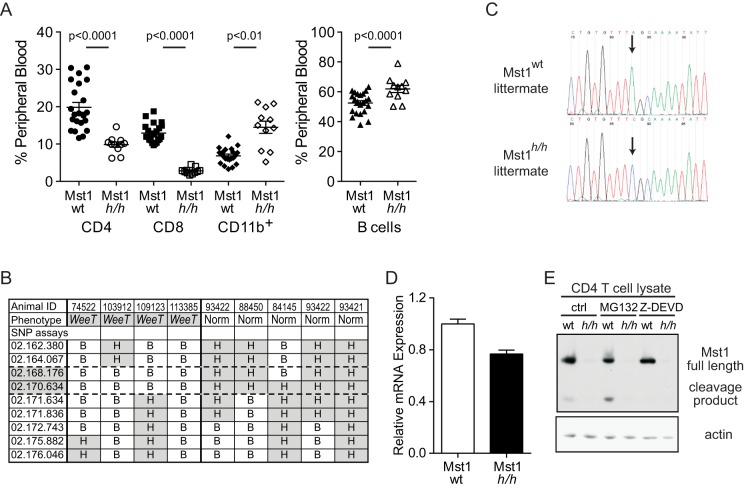
*WeeT* mice have reduced peripheral CD4 and CD8 T cells due to *Mst1* deficiency. **A**) Representation of CD4 and CD8 T cells, CD11b^+^ and B cells in the peripheral blood of *Mst1^wt^* and *Mst1^h/h^* mice. **B**) Inheritance of homozygous C57BL/6 (B), 129Sv/ImJ (C) or heterozygous (H) SNPs in F2 mice generated by crossing *Mst1^h/h^* mice from the original C57BL/6 background to 129Sv/ImJ. Genetic mapping of T-lymphopenic (*WeeT*) and normal mice isolated a 4.5 Mb region on chromosome 2 harboring the causative mutation. **C**) *Mst1^h/h^* mice harbor an A to C transversion in exon 5 of the *Mst1* gene, resulting in change of Leu_157_ within the Mst1 kinase C-lobe to Arg (L_157_R). **D**) Similar abundance of *Mst1* transcripts in wt and *Mst1^h/h^* T cells. **E**) *Mst1^h/h^* T cells have reduced Mst1 protein levels in the presence or absence of proteosome (MG132) or caspase-3 inhibitors (Z-DEVD).

In an ENU-mutagenesis screen for genetic mutations resulting in T cell lymphopenia, we identified one pedigree, named *WeeT* (*Mst1^h/h^*, see below) with reduced proportions of conventional CD4 and CD8 αβ T cells in the peripheral blood (Fig. 1A). Approximately 25% of G3 progeny were lymphopenic, indicative of a single recessive mutation. CD11b^+^ myeloid and B220^+^ B cell proportions were mildly increased. *WeeT* mice were out-crossed to 129Sv/ImJ mice and F2 progeny were used to map the causative mutation by correlating phenotype and inheritance of C57BL/6 (B), 129Sv/ImJ (C) or both (H, heterozygous) single nucleotide polymorphisms (SNPs) using a SNP panel [31], [35]. A perfect genotype-phenotype correlation identified a 4.5 Mb region on chromosome 2 (Fig. 1B). Deep sequencing of genomic DNA following enrichment for exons in the 4.5 Mb region revealed an A to C transversion in exon 5 of the *Mst1* gene (Fig. 1C), resulting in substitution of Leu at amino acid position 157 with Arg (L_157_R). This mutation did not disrupt *Mst1* transcript levels (Fig. 1D). Instead, we observed a loss of Mst1 protein, in either its full-length or caspase-cleaved form that did not recover following short-term treatment (4 hours) with proteosome (MG132) or caspase-3 (Z-DEVD) inhibitors (Fig. 1E). Thus, we conclude that the *WeeT* mutation caused Mst1 protein loss similar to conventionally-targeted *Mst1* deficient mice, and hereafter, we refer to homologous mutant *WeeT* mice as Mst1^h/h^.

### *Mst1^h/h^* mutation abrogates Mst1 function *in vivo*

To determine whether Mst1^h/h^ resulted in a similar immune phenotype as *Mst1* knockout mice, we phenotypically characterized *Mst1^h/h^* mice. Similar to conventional *Mst1* knockout mice, we observed a 3- and 5-fold reduction in splenic CD4 and CD8 T cells in *Mst1^h/h^* versus wt littermate mice (Fig. S1). As previously reported for *Mst1*-deficient mice [4], [6], an accumulation of CD4 and CD8 single positive (SP) thymocytes was observed, particularly affecting HSA^low^CD69^neg^ emigration-ready SP cells (Fig. S1). Defects in T cell development and function are often masked or underrepresented when analyzed on a normal C57BL/6 background due to the low efficiency of T cell development or compensation within the TCR repertoire. Transgenic expression of a single TCR allows better visualization of defects on a monoclonal population of T cells. Thus, we crossed the 5C.C7 TCR transgene onto the Mst1^h/h^ background. An even greater decrease in peripheral and concomitant increase in thymic CD4 T cells was observed in *Mst1*-deficient mice bearing the TCR transgene, 5C.C7 (Fig. S2). Thus, the L_157_R mutation in the Mst1 led to a phenotype similar to complete *Mst1* knockout.

### LFA-1 engagement compensates for *Mst1* deficiency in CCL19-induced T cell polarization

**Figure 2:**
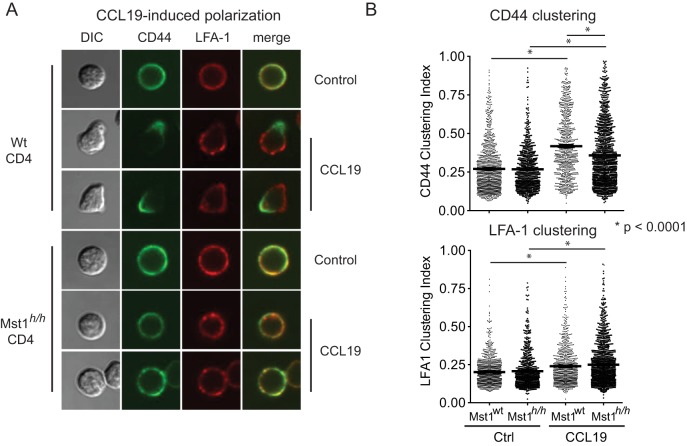
Mst1 is required for integrin-independent T cell polarization. **A**) Wt and *Mst1^h/h^* CD4 T cells were stimulated with 100 ng/mL CCL19 in PBS for 30 minutes. Polarization of CD44 to the uropod and LFA-1 distribution were visualized by confocal microscopy. **B**) Computational scoring of CD44 and LFA-1 clustering on wt and *Mst1^h/h^* CD4 T stimulated with 100 ng/mL CCL19 in PBS prior to fixation and staining for LFA-1 and CD44 expression. Student's t-test was performed to compare clustering efficiency for Mst1^wt^ and Mst1^h/h^ T cells.

T cells respond to chemotactic cues by spatially redistributing cell surface receptors and signaling molecules to facilitate migration. Polarization of chemokine receptors to the leading edge and accumulation of receptors including CD44 in the uropod allow more efficient directional movement [36]. *Mst1*-deficient T cells are defective in polarization [5], [6]. To better understand how Mst1-loss disrupts polarization, we quantified polarization by calculating CD44 receptor clustering in >800 individual T cells based on a modified method [34]. Consistent with previous studies, the degree of CD44 clustering decreased significantly in Mst1^h/h^ T cells stimulated with the chemokine CCL19 for 30 minutes (Fig. 2A, B). Surprisingly, we did not observe a gross difference between wt and *Mst1*-deficient cells in clustering of global LFA-1 receptors (Fig. 2A, B). These quantitative data confirmed that chemokine-induced T cell polarization is Mst1 dependent.

**Figure 3:**
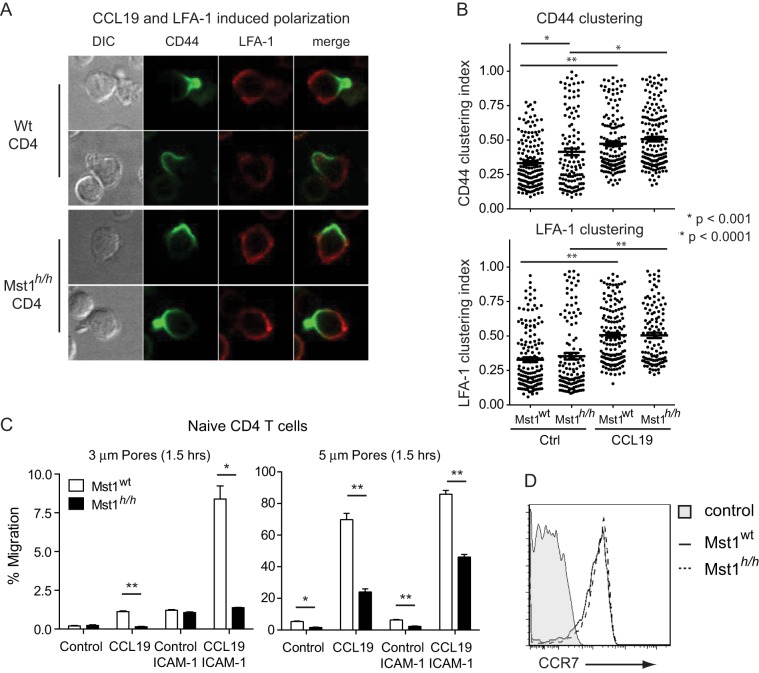
Mst1 is dispensable for integrin-dependent T cell polarization by required for CCL19-induced migration. **A**) Wt and *Mst1^h/h^* CD4 T cells were seeded into slide chambers pre-coated with 100 ng/mL ICAM-1-Fc prior to stimulation with CCL19. Polarization of CD44 to the uropod in comparison to LFA-1 expression was visualized by confocal microscopy. **B**) Computational scoring of CD44 and LFA-1 clustering during live imaging of wt and *Mst1^h/h^* CD4 T cells on ICAM-1 coated chamberslides stimulated for 30 minutes with 100 ng/mL CCL19 in presence of 0.08 ng/mL Alexa647-anti-CD11a/LFA-1 (M17/4) and Alexa488-anti-CD44. For each time point, 99–166 individual cells were analyzed for receptor clustering. Student's t-test was performed to compare clustering efficiency for Mst1^wt^ and Mst1^h/h^ T cells. **C**) Transmigration of purified wt and *Mst1^h/h^* CD4 T cells in response to 100 ng/mL CCL19 through 3 μm or 5 μm pores pre-coated with BSA or ICAM-1 Fc. Data is displayed as mean ± SEM of triplicate samples in a single experiment representative of 3–5 independent experiments. Student's t-test was performed to compare migration efficiency for Mst1^wt^ and Mst1^h/h^ T cells, * p<0.002, ** p<0.0001. **D**) CCR7 expression was determined by flow cytometry.

Chemokine-induced T cell polarization can be enhanced by outside-in integrin signaling, which uses distinct signaling pathways to regulate the actin cytoskeleton [12] [37]. To determine whether Mst1 also plays a role in the outside-in integrin signaling, wt and *Mst1^h/h^* CD4 T cells were seeded onto ICAM-1 coated chamber slides prior to CCL19 addition. Polarization was monitored by time-lapse imaging in the presence of low concentrations of fluorescently labeled antibodies against LFA-1 and CD44. Low-dose antibody addition neither inhibited adhesion to ICAM-1 nor led to differences in the migratory behavior compared to unstained wt or *Mst1^h/h^* CD4 T cells (Fig. S3). Interestingly, ICAM-1 engagement of LFA-1 leads to normal CCL19-induced polarization of *Mst1^h/h^* CD4 T cells, and enhancement of LFA-1 clustering in both wt and *Mst1^h/h^* CD4 T cells (Fig. 3A, B). This indicates that Mst1 is required for T cell polarization in response to chemokine signaling but dispensable for polarization induced by outside-in integrin signaling.

### Mst1 is important for cellular contractility

To directly investigate the requirements for Mst1 in chemokine-induced migration, we first assessed the ability of CCL19 to induce CD4 T cell migration through transwell membranes. Broad defects in migration can be detected by evaluating migration through 5 μm pores. Specific defects in cellular contractility can be detected by further evaluating migration through 3 μm pores [27]. CCL19 induced a 8-fold increase in the migration of wt CD4 T cells through either 3 or 5 μm transwell pores compared to chemokine-free controls (Fig. 3C). In contrast, CCL19 induced only a 1.6-fold increase in the migration of *Mst1^h/h^* CD4 T cells through 5 μm pores and no detectable migration through 3 μm pores despite normal expression of CCR7 (Fig. 3C, D). This general migration defect is consistent with the inability of *Mst1^h/h^* T cells to establish cell polarity in response to chemokine stimulation alone (Fig. 2A, B).

To further validate the observation that Mst1 is not a component of the integrin outside-in pathway (Fig. 3A, B), we compared the ability of wt and *Mst1^h/h^*T cells to migrate across transwells coated with ICAM-1. As expected, the presence of ICAM-1 readily and strongly enhanced CCL19-induced chemotaxis of wt CD4 T cells (Fig. 3C). LFA-1 engagement especially promoted migration of wt T cells through 3 μm pores, which are less than half the cell's diameter. This is consistent with the observation that migration through 3 μm pores is particularly dependent on myosin-mediated contractility and is facilitated by integrins [37].

Interestingly, ICAM-1 enhanced migration of *Mst1^h/h^* T cells through 5 μm pores in response to CCL19 (Fig. 3C). This is likely due to the ability of ICAM-1 to induce LFA-1-dependent polarization of *Mst1^h/h^* T cells (Fig. 3A, B). However, ICAM-1 was unable to promote CCL19-induced migration through 3 μm pores compared to chemokine-free, ICAM-1 only controls (Fig. 3C). These findings indicate that LFA-1 outside-in signaling can partially compensate for *Mst1* deficiency in promoting T cell migration through non-constraining pores; however, there is a strict requirement for Mst1 in T cell migration that requires cellular contractility.

### Mst1 regulates Myosin IIa localization

**Figure 4:**
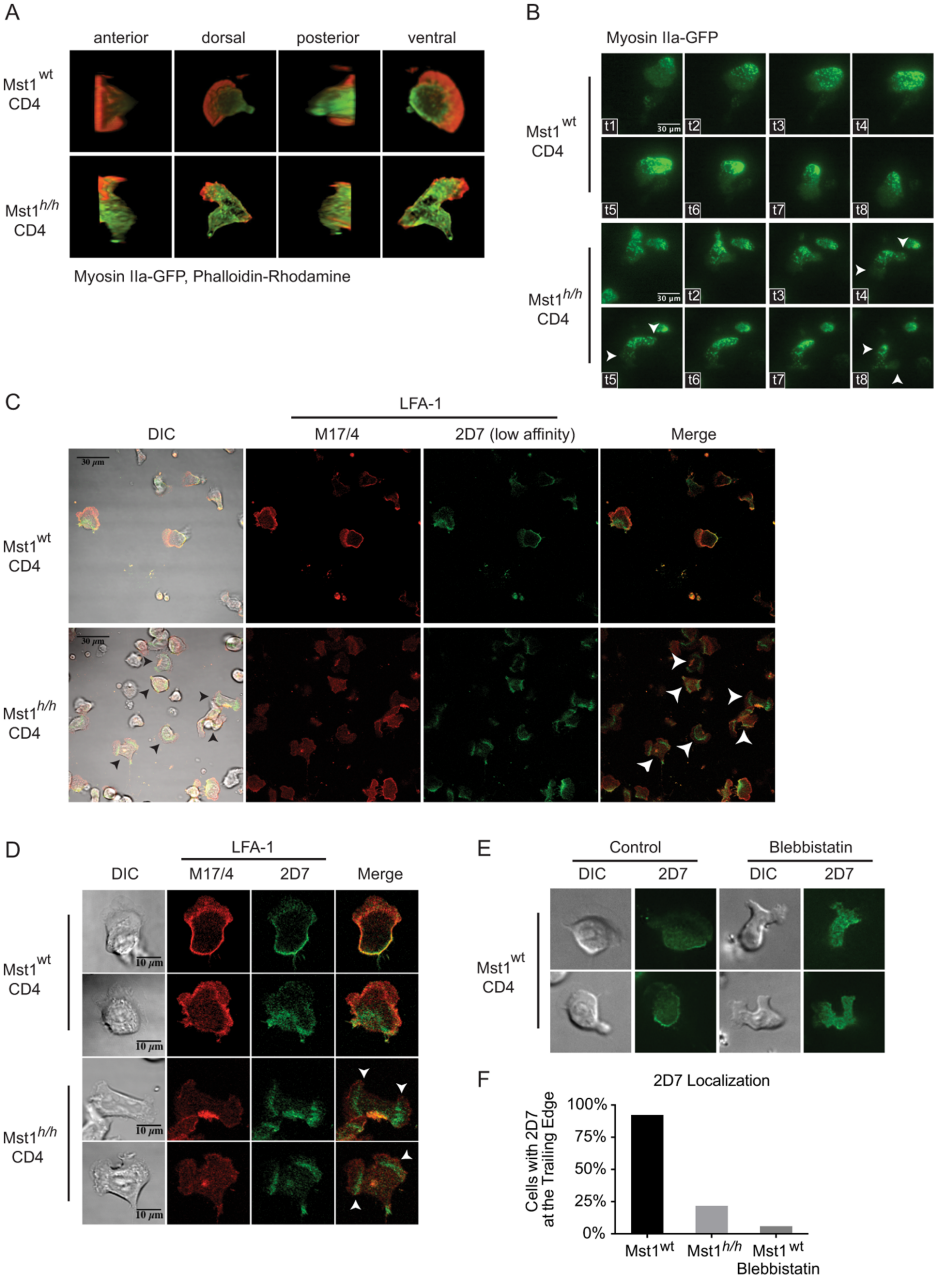
Mst1 regulates Myosin IIa localization and is required for partitioning of low and higher affinity LFA-1 molecules. **A**) Wt and *Mst1^h/h^* CD4 T cells expressing Myosin IIa-GFP were seeded into slide chambers pre-coated with 1 μg/mL ICAM-1-Fc and stimulated with CCL19 prior to fixation and staining of F-actin with Rhodamine-phalloidin. Three-dimensional image reconstructions from z-stacks of confocal micrographs are displayed. **B**) Wt and *Mst1^h/h^* CD4 T cells expressing Myosin IIa-GFP were visualized by live TIRF microscopy. Arrows indicate bipolar morphology. Data are representative of 2 individual experiments with 150 cells per genotype. **C, D**) Wt and *Mst1^h/h^* CD4 T cells were stimulated as above and stained with 2D7 (anti-low affinity CD11a/LFA-1, green) and M17/4 (anti-CD11a/LFA-1, red). **E**) Wt CD4 T cells stimulated as above with or without Blebbistatin treatment were stained with 2D7 and visualized by immunofluorescence. **F**) Quantification of cells with 2D7 localization at the trailing edge of untreated or Blebbistatin-treated Wt and Mst1^h/h^ CD4 T cells (data are representative of 2–3 individual experiments, n = 13–23).

To determine how Mst1 regulates cellular contractility, we performed live differential interference contrast (DIC) imaging of wt and *Mst1^h/h^* CD4 T cells migrating on ICAM-1-coated surfaces. Over time, a fraction of *Mst1^h/h^* CD4 T cells (Videos S3, S4) but no wt cells formed long uropods (Videos S1, S2). These elongated cells represent a subpopulation of *Mst1^h/h^* CD4 T cells with severe defects in contraction, similar in scale to pharmacologic inhibition of ROCK, an activator of Myosin IIa at the trailing edge [37]. To investigate Myosin IIa directly, we used confocal microscopy to visualize the localization of Myosin IIa-GFP fusion protein and F-actin by staining migrating wt and *Mst1^h/h^* CD4 T cells with phalloidin. Three-dimensional reconstruction of z-axis serial confocal micrographs of wt CD4 T cells allowed us to visualize the lamellipodia, the lamellae with dorsal and lateral membrane ruffles, the trailing edge of the membrane contacting the substratum, and the upwards-pointing uropod. While F-actin is sparse in the uropod, Myosin IIa is particularly enriched in the membrane extending from the trailing edge towards and into the uropod of wt cells (Fig. 4A, Video S5). Myosin IIa co-localized with the actin cytoskeleton at sites of membrane ruffling and at the trailing edge, as observed in three-dimensional reconstruction of wt CD4 T cells (Fig. 4A, Video S5). Three-dimensional reconstruction of *Mst1^h/h^* CD4 T cell images revealed several abnormalities. The leading edge of *Mst1^h/h^* CD4 T cells did not form a classic fan-like lamellipodium. Membrane ruffles were observed in the leading edge rather than in the dorsal lamellae (Fig. 4A, Video S6). Similar to wt cells, Myosin IIa co-localized with F-actin in the trailing edge. However, instead of extending predominantly into the uropod, Myosin IIa was diffusely localized throughout the midbody and lamellae of *Mst1^h/h^* CD4 T cells (Fig. 4A, Video S6). The dynamics of Myosin IIa-GFP were also assessed by live TIRF imaging to visualize Myosin IIa near the ventral membrane that contacts the substratum. In wt T cells, Myosin IIa clusters were sparsely observed in the midbody but enriched in the posterior membrane during migration (Fig. 4B, Video S7). Although Myosin IIa clusters were observed in the posterior membrane of *Mst1^h/h^* T cells, it was also present in the midbody and extended into multipolar anterior protrusions (Fig. 4B, Video S8). These data indicate that Myosin IIa localization was dysregulated in the absence of Mst1, resulting in a defect in T cell contraction.

### Mst1-dependent Myosin IIa activity controls the spatial distribution of low and higher affinity LFA-1

Myosin IIa contraction regulates LFA-1 adhesion and de-adhesion [21], [22], [37]. Different affinity LFA-1 conformations are spatially segregated within the membrane of migrating T cells. Affinity-specific antibodies have revealed that LFA-1 molecules at the leading edge and midbody are in the intermediate and high affinity conformations respectively while uropodal and trailing edge LFA-1 molecules are of low affinity [16]–[18]. To determine if the mis-localization of Myosin IIa observed in *Mst1*-deficient cells affects the distribution of different affinity LFA-1 molecules, wt and *Mst1^h/h^* CD4 T cells were stained with the antibody clone 2D7, which recognizes low affinity LFA-1 [38]. As previously published, low affinity LFA-1 was restricted to the trailing edge of wt CD4 T cells (Fig. 4C, D). Unfortunately, antibodies that specifically recognize intermediate and high affinity LFA-1 are unavailable for mouse cells. However, co-staining T cells with limiting amounts of the pan-affinity specific LFA-1 antibody, M17/4 together with 2D7 allowed preferential detection of higher (intermediate and high) affinity LFA-1 by M17/4 (Fig. 4C, D). Dual staining with M17/4 and 2D7 revealed that wt cells showed enriched distribution of higher affinity LFA-1 in the midbody behind the leading edge (Fig. 4C, D). In contrast, in many *Mst1^h/h^* CD4 T cells, low affinity LFA-1 was distributed inappropriately to the lamellae, an actin-rich region behind the leading edge. Moreover, a considerable number of *Mst1^h/h^* CD4 T cells generated two leading edge protrusions, with both lamellae containing mis-localized low affinity LFA-1 (Fig. 4C, D). Higher affinity LFA-1 was also mis-localized in multipolar cells, generally to the leading or trailing edges. Thus, we conclude that loss of Mst1 disrupts the spatial organization of low and higher affinity LFA-1 and suggest that this defect significantly contributes to the well-established adhesion defects observed of *Mst1*–deficient T cells.

To determine whether inhibition of Mysoin IIa activity also disrupts the localization of low affinity LFA-1 in Mst1^h/h^ cells, wt CD4 T cells were treated with Blebbistatin, an inhibitor of myosin ATPase activity [39]. Low affinity LFA-1 distribution to the trailing edge was disrupted in both Blebbistatin-treated wt and untreated Mst1^h/h^ T cells (Fig. 4D, E, F). However, Blebbistatin-treated wt cells exhibited a broad distribution of low affinity LFA-1 (Fig. 4E) while Mst1^h/h^ cells showed inappropriate LFA-1 localization to the lamella (Fig. 4D). This disparity may be due to partial compensation by Mst2 or other unknown factors. However, both Mst1^h/h^ cells and Blebbistatin-treated wt cells could form two leading edges (Fig. 4D, E). Together, these data support a novel regulatory role for Mst1 in coordinating Myosin IIa contractility to facilitate the appropriate distribution of low and higher affinity integrins during T cell migration.

## Discussion

Together, our data demonstrates that Mst1 regulates T cell polarization and promotes progressive integrin-dependent T cell migration through control of Myosin IIa activity. Visualization of Myosin IIa and F-actin localization using confocal and TIRF microscopy allowed us to identify a role for Mst1 in restricting Myosin IIa localization to dorsal membrane ruffles, the trailing edge membrane, and the uropod. Moreover, we show that Myosin IIa regulates the spatial distribution of low and high affinity LFA-1 in migrating T cells. *Mst1* deficiency or Myosin II inhibition resulted in the establishment of multipolar cells, elongated uropods and deregulated localization of low affinity LFA-1.

Determining how Mst1 regulates Myosin IIa will be an important goal for future studies. Precise control of contraction is mediated by phosphorylation of multiple sites within both Myosin light and heavy chains [19]. Normal levels of di-phosphorylated-Myosin Light Chain (MLC-T_18_S_19_) in *Mst1^h/h^* T cells suggest that Mst1 is not required to regulate MLC at these sites (Fig. S4). However, It will be important to evaluate a role for Mst1 phosphorylation of Myosin at other sites and of more proximal signaling proteins in future experiments.

Another possible mechanism is if Mst1 controls LFA-1 anchoring to the actinomyosin network. LFA-1 association with the actin cytoskeleton is via binding to Talin [40] and coincides with association of LFA-1 with Myosin IIa [22]. Talins are recruited to the CD18 cytoplasmic domain of LFA-1 by RIAM, an adapter protein that associates with Kindlin and Mst1 [40]. It will be important in future studies to assess the necessity for Mst1 kinase activity or adapter function in RIAM-dependent anchoring of LFA-1 to the actin cytoskeleton. While multiple components of the focal adhesion complex can be phosphorylated [41], it remains to be determined whether any are Mst1 substrates and how phosphorylation affects integrin association with the actinomyosin network.

Myosin IIa-mediated contraction is also required for antigen-dependent responses. Although interstitial migration in the lymph node is integrin-independent [42], T cells rely on Myosin IIa-dependent contraction to squeeze through narrow gaps [23], [24], [43]. As they migrate, they form transient immune kinapses with antigen presenting cells [44]. Upon high affinity binding between the T cell receptor and its cognate peptide-MHC antigen, a stable, long lasting immune synapse forms. The immune synapse is spatially organized into concentric regions with TCRs accumulating in the central supramolecular activation cluster (cSMAC) surrounded by LFA-1 in the peripheral SMAC. LFA-1 recruitment to the pSMAC and subsequent delivery of the microtubule organizing center (MTOC) to the synapse are dependent on Myosin IIa [45], [46]. While much is known about MTOC delivery [47], it less clear how Myosin delivers LFA-1 to the pSMAC. Interestingly, Mst1 is activated following TCR stimulation [48] and is required for stable immune synapse formation [5]. It remains to be determined if LFA-1 recruitment to the pSMAC is also regulated by Mst1-directed Myosin IIa activity.

In summary, we have identified a new requirement for Myosin IIa in controlling the spatial distribution of low and high affinity LFA-1 and have demonstrated a requirement for Mst1 in controlling Myosin IIa localization and activity during T cell migration. By advancing our insight into the molecular mechanisms controlling integrin function, T cell contractility, polarization and migration, our findings help to elucidate the distinct cellular defects that cause the primary immunodeficiency resulting from Mst1 dysfunction.

## Supporting Information

Figure S1 **Reduced naïve peripheral T cells and increased mature thymocytes in Mst1^h/h^ mice. A**) Representation and **B**) total cell numbers of splenic naïve (CD44low) and effector/memory (CD44high) T cells and thymic populations. CD4SP and CD8SP thymocytes expressing high levels of αβTCR were further analyzed for maturity based on differential expression of CD69 and HSA. Less mature SP thymocytes were HSA^+^CD69^+^, while more mature thymocytes were HSA^low^CD69^neg^.
(EPS)

Figure S2 **Reduced peripheral 5CC7 TCR tg Mst1** ***^h/h^*** **CD4 T cells due to a defect in egress of mature thymocytes. A**) Splenic 5CC7 TCR transgenic T cells were assessed for prior activation based on CD62L expression. **B**) Thymic profiles of wt and Mst1 *^h/h^* 5CC7 TCR tg mice including delineation of less mature (HSA^+^CD69^+^) and more mature (HSA^low^CD69^neg^) clonotypic CD4SP thymocytes.
(EPS)

Figure S3 **There is no difference in the migration behavior of wt CD4 T cells in the presence of absence of M17/4 antibody.** Wt CD4 T cells were seeded into slide chambers pre-coated with 1 µg/mL ICAM-1-Fc and stimulated with CCL19 and imaged in real time in the **A**) absence or **B**) presence of 0.08 ng/ml M17/4 anti-LFA-1 antibody. Each dot/line represents a single cell. Cell distance and direction were tracked and graphed. Mean distance traveled and mean velocities are indicated.
(EPS)

Figure S4 **Levels of di-phosphorylated Myosin Light Chain (T18S19) determined by analyzing fluorescent micrographs of wt and Mst1^h/h^ CD4 T cells stimulated with CCL19 in ICAM-1 coated chamber slides.**
(EPS)

Video S1 **Migrating wt CD4 T cells in ICAM-1 coated chamber slides in response to CCL19 (example 1).**
(MOV)

Video S2 **Migrating wt CD4 T cells in ICAM-1 coated chamber slides in response to CCL19 (example 2).**
(MOV)

Video S3 **Migrating Mst1^h/h^ CD4 T cells in ICAM-1 coated chamber slides in response to CCL19 (example 1).**
(MOV)

Video S4 **Migrating Mst1^h/h^ CD4 T cells in ICAM-1 coated chamber slides in response to CCL19 (example 2).**
(MOV)

Video S5 **Three-dimensional image reconstruction from z-stacks of confocal micrographs of representative Wt CD4 T cell expressing Myosin IIa-GFP (green) were seeded into slide chambers pre-coated with 1 μg/mL ICAM-1-Fc and stimulated with CCL19 prior to fixation and staining of F-actin with Rhodamine-phalloidin (red).**
(AVI)

Video S6 **Three-dimensional image reconstruction from z-stacks of confocal micrographs of representative** ***Mst1^h/h^*** **CD4 T cell expressing Myosin IIa-GFP (green) were seeded into slide chambers pre-coated with 1 μg/mL ICAM-1-Fc and stimulated with CCL19 prior to fixation and staining of F-actin with Rhodamine-phalloidin (red).**
(AVI)

Video S7 **TIRF imaging of migrating wt CD4 T cells expressing Myosin II-GFP.**
(AVI)

Video S8 **TIRF imaging of migrating Mst1^h/h^ CD4 T cells expressing Myosin II-GFP.**
(AVI)
